# Randomized Phase 3 Trial of Ruxolitinib for COVID-19–Associated Acute Respiratory Distress Syndrome*

**DOI:** 10.1097/CCM.0000000000005682

**Published:** 2022-10-13

**Authors:** Lindsay Rein, Karel Calero, Ronak Shah, Charles Ojielo, Kristin M. Hudock, Saba Lodhi, Farid Sadaka, Shashi Bellam, Christopher Palma, David N. Hager, Jeannie Daniel, Richard Schaub, Kevin O’Hayer, Nicole M. Theodoropoulos

**Affiliations:** 1 Department of Medicine, Duke University Medical Center, Durham, NC.; 2 Department of Internal Medicine, Pulmonary Critical Care and Sleep Medicine, University of South Florida, Tampa, FL.; 3 Division of Pulmonary and Critical Care Medicine, Hackensack Meridian School of Medicine, Hackensack, NJ.; 4 Section of Critical Care Medicine, Aurora St. Luke’s Medical Center, Milwaukee, WI.; 5 Division of Pulmonary, Critical Care & Sleep Medicine, University of Cincinnati School of Medicine, Cincinnati, OH.; 6 Pulmonology, Confluence Health, Wenatchee, WA.; 7 Trauma and Neurologic Intensive Care Unit, Mercy Hospital, St. Louis, MO.; 8 Department of Medicine, NorthShore University HealthSystem, Evanston, IL.; 9 Department of Medicine, University of Rochester Medical Center, Rochester, NY.; 10 Department of Medicine, Johns Hopkins University, Baltimore, MD.; 11 Clinical Development, Incyte Corporation, Wilmington, DE.; 12 Division of Infectious Diseases & Immunology, UMass Memorial Medical Center, Worcester, MA.

**Keywords:** COVID-19, Janus kinase inhibitors, randomized controlled trial, respiration, artificial, respiratory distress syndrome, severe acute respiratory syndrome coronavirus

## Abstract

Evaluate the safety and efficacy of the Janus kinase (JAK)1/JAK2 inhibitor ruxolitinib in COVID-19–associated acute respiratory distress syndrome requiring mechanical ventilation.

**DESIGN:**

Phase 3 randomized, double-blind, placebo-controlled trial Ruxolitinib in Participants With COVID-19–Associated Acute Respiratory Distress Syndrome Who Require Mechanical Ventilation (RUXCOVID-DEVENT; NCT04377620).

**SETTING:**

Hospitals and community-based private or group practices in the United States (29 sites) and Russia (4 sites).

**PATIENTS:**

Eligible patients were greater than or equal to 12 years old, hospitalized with severe acute respiratory syndrome coronavirus 2 infection, and mechanically ventilated with a Pao_2_/Fio_2_ of less than or equal to 300 mm Hg within 6 hours of randomization.

**INTERVENTIONS:**

Patients were randomized 2:2:1 to receive twice-daily ruxolitinib 15 mg, ruxolitinib 5 mg, or placebo, each plus standard therapy.

**MEASUREMENTS AND MAIN RESULTS:**

The primary endpoint, 28-day mortality, was tested for each ruxolitinib group versus placebo using a mixed-effects logistic regression model and one-tailed significance test (significance threshold: *p* < 0.025); no type 1 error was allocated to secondary endpoints. Between May 24, 2020 and December 15, 2020, 211 patients (age range, 24–87 yr) were randomized (ruxolitinib 15/5 mg, *n* = 77/87; placebo, *n* = 47). Acute respiratory distress syndrome was categorized as severe in 27% of patients (58/211) at randomization; 90% (190/211) received concomitant steroids. Day-28 mortality was 51% (39/77; 95% CI, 39–62%) for ruxolitinib 15 mg, 53% (45/85; 95% CI, 42–64%) for ruxolitinib 5 mg, and 70% (33/47; 95% CI, 55–83%) for placebo. Neither ruxolitinib 15 mg (odds ratio, 0.46 [95% CI, 0.201–1.028]; one-sided *p* = 0.029) nor 5 mg (odds ratio, 0.42 [95% CI, 0.171–1.023]; one-sided *p* = 0.028) significantly reduced 28-day mortality versus placebo. Numerical improvements with ruxolitinib 15 mg versus placebo were observed in secondary outcomes including ventilator-, ICU-, and vasopressor-free days. Rates of overall and serious treatment-emergent adverse events were similar across treatments.

**CONCLUSIONS:**

The observed reduction in 28-day mortality rate between ruxolitinib and placebo in mechanically ventilated patients with COVID-19–associated acute respiratory distress syndrome was not statistically significant; however, the trial was underpowered owing to early termination.

Severe acute respiratory syndrome coronavirus 2 (SARS-CoV-2) has caused a global health emergency, with many people developing clinically significant COVID-19, and 20% requiring hospitalization (1-3). One third of hospitalized patients develop acute respiratory distress syndrome (ARDS), a life-threatening inflammatory lung condition characterized by loss of aerated tissue, severe hypoxemia, and increased dead space (4, 5). Despite standard use of lung-protective ventilation strategies and prone positioning, patients with COVID-19–associated ARDS requiring invasive mechanical ventilation have poor outcomes (4, 6–8). Among these patients, only dexamethasone plus interleukin (IL)-6 inhibition or Janus kinase (JAK) inhibition has been shown to improve survival, but mortality remains high (9-11). Therefore, an unmet need remains for additional effective therapies.

COVID-19 is associated with aberrant cytokine signaling, including overactivation of the JAK/signal transducers and activators of transcription (STAT) pathway (12). Several cytokines that activate JAK/STAT signaling, including interferon-γ, granulocyte-macrophage colony-stimulating factor, IL-2, and IL-6, are overexpressed in patients with COVID-19 (13, 14), and inhibiting some of these inflammatory mediators is associated with clinical improvement compared with standard of care (10, 15, 16). The IL-6 inhibitor tocilizumab has received emergency use authorization; however, evidence from randomized trials of patients with severe disease is mixed (10, 17). Because the COVID-19–associated cytokine storm involves several cytokines in addition to IL-6, blocking multiple inflammatory mediators via JAK/STAT inhibition is a rational therapeutic approach (12). Furthermore, the inflammatory response of COVID-19 exhibits a similar macrophage-derived cytokine profile as hemophagocytic lymphohistiocytosis (18), which is responsive to the selective JAK1/JAK2 inhibitor ruxolitinib (19, 20). Several reports have provided proof of concept for JAK inhibition for the treatment of critically ill patients with COVID-19 (11, 21-24); however, only one exploratory study has investigated a JAK inhibitor (baricitinib) specifically in patients with COVID-19–associated ARDS requiring mechanical ventilation (11). The objective of this study was to evaluate the effects on mortality and in-hospital outcomes (ventilator-, ICU-, oxygen-, vasopressor-, and hospital-free days) as well as the safety of the investigational agent ruxolitinib in mechanically ventilated patients with COVID-19–associated ARDS.

## METHODS

### Study Design and Patients

Ruxolitinib in Participants With COVID-19–Associated ARDS Who Require Mechanical Ventilation (RUXCOVID-DEVENT; ClinicalTrials.gov‚ NCT04377620) was a double-blind, randomized, placebo-controlled, multicenter phase 3 trial assessing ruxolitinib in mechanically ventilated patients with COVID-19–associated ARDS. Eligible patients were at least 12 years old, hospitalized with SARS-CoV-2 infection (confirmed ≤ 3 wk before randomization), mechanically ventilated with arterial oxygen partial pressure/fractional inspired oxygen (Pao_2_/Fio_2_) of less than or equal to 300 mm Hg within 6 hours of randomization, and had bilateral pulmonary infiltrates on x-ray or CT chest scan. Exclusion criteria included suspected active uncontrolled bacterial, fungal, viral, or other infection (besides COVID-19); being unlikely to survive longer than 24 hours from randomization per investigator judgment; receiving extracorporeal membrane oxygenation; or receiving JAK inhibitor treatment within 30 days of randomization.

The central institutional review board (WCG Western Institutional Review Board) approved the study (approval number 20201074). Informed consent was obtained from patients’ healthcare proxy per an institutionally approved method before study treatment initiation. The trial was performed in accordance with the principles embodied by the Declaration of Helsinki with adherence to the trial protocol and the International Council for Harmonisation Guideline for Good Clinical Practice.

### Procedures

Interactive response technology was used to block randomize patients 2:2:1 to ruxolitinib 15 mg twice a day (BID)‚ ruxolitinib 5 mg BID‚ or matching dual-dose placebo tablets. Given the expected high mortality rate and anticipation of a treatment effect, the 2:2:1 ratio was employed to allow for ~80% of patients to receive ruxolitinib. Randomization was stratified by ARDS severity (mild/moderate [Pao_2_/Fio_2_ > 100–300 mm Hg] vs severe [Pao_2_/Fio_2_ ≤ 100 mm Hg]) and investigational site. Treatment identity was concealed by the use of identical packaging, administration schedule, appearance, taste, and color. The study statistician and programming team were unblinded before database lock (i.e., no further changes to trial data allowed) after statistical analysis plan finalization to confirm that randomization codes had been applied correctly by the independent statistician; otherwise, patients, investigators, and the study sponsor remained blinded to treatment from the time of randomization until database lock. A 1-day screening period was followed by a 28-day follow-up period (**eFig. 1**, http://links.lww.com/CCM/H210). The initial treatment period lasted 14 days, after which patients could continue to receive ruxolitinib or placebo treatment for an additional 14 days if the benefit and risk were deemed appropriate in the opinion of the site investigator in consultation with the blinded medical monitor on Day 15, without knowledge of study drug identity. Crossover between groups was not permitted. Safety follow-up assessments were conducted 28 ± 3 days after the last dose of trial treatment. Trial treatment was administered via an enteric feeding tube or orally, as clinically appropriate.

### Outcomes

The primary endpoint was 28-day mortality, defined as the proportion of patients who died due to any cause through Day 29. Secondary efficacy endpoints included in-hospital outcomes (number of ventilator-free days, ICU-free days, oxygen-free days, vasopressor-free days, and hospital-free days) at day 29; change in Sequential Organ Failure Assessment (SOFA) score from baseline to days 3, 5, 8, 11, 15, and 29; and clinical status using the World Health Organization (WHO) nine-point ordinal scale at days 15 and 29 (**eTable 1**, http://links.lww.com/CCM/H210) (25). The safety endpoint was the number and proportion of patients with treatment-emergent adverse events (TEAEs; any adverse event either reported for the first time or worsening of a pre-existing event after first dose of study drug until 31 days after the last dose of study drug) and serious TEAEs, including clinically significant changes in laboratory measures and vital signs. Lack of efficacy and failure of expected pharmacologicaction (e.g.‚ disease progression) were not reported as TEAEs. Additional details including timing of assessments are provided in the **Supplemental Digital Content** (http://links.lww.com/CCM/H210).

### Statistical Analyses

The primary endpoint was tested for each ruxolitinib group using a mixed-effects logistic regression model including treatment (ruxolitinib vs placebo) and ARDS severity (severe vs mild/moderate) as fixed effects and investigational site as a random intercept effect. Patients lost to follow-up before day 29 were not evaluable for the primary analysis but were included in a sensitivity analysis in which they were imputed as deaths. A sample size of 500 patients (pairwise comparison of 200 randomized to each ruxolitinib group and 100 to placebo) was initially planned to achieve approximately 83% power to detect a statistically significant effect of ruxolitinib with a nominal one-sided type I error of 1.44% (Dunnett’s procedure), assuming a mortality rate of 40% for ruxolitinib versus 60% for placebo based on limited mortality data emergent early in the pandemic (8, 26). Owing to slow enrollment and an expected decline in U.S. cases with vaccine uptake, preliminary negative results from a separate trial of ruxolitinib in nonintubated hospitalized patients with COVID-19–associated cytokine storm (NCT04362137), and approval and commercial availability of another JAK1/JAK2 inhibitor (baricitinib) for this indication, the sponsor prematurely halted enrollment into RUXCOVID-DEVENT; final analyses were performed using all randomized patients (*n* = 211). The type I error level for the primary endpoint in this analysis was one-sided 2.5%; a fixed-sequence testing procedure was used to test the ruxolitinib 15 mg twice-daily group versus placebo first, and if significant, the 5 mg twice-daily group was tested. Use of one-sided type I error was based on the one-sided hypothesis that ruxolitinib added to standard of care would reduce 28-day mortality versus placebo plus standard of care. Two-sided CIs were presented for estimation of the mortality rate in both directions. Secondary efficacy variables were tested at the 0.05 level using a two-sided test; no type 1 error was allocated. Post hoc analyses of the primary endpoint for each ruxolitinib group in the U.S.-only population and pooled ruxolitinib treatment regimens in the full population were performed using similar methodology to the primary analysis.

The intention-to-treat population (all randomized patients) was used for analysis of summary demographics, baseline characteristics, patient disposition, and all efficacy outcomes unless otherwise noted. The safety population included all patients who received at least one dose of trial drug.

## RESULTS

**Figure 1.**
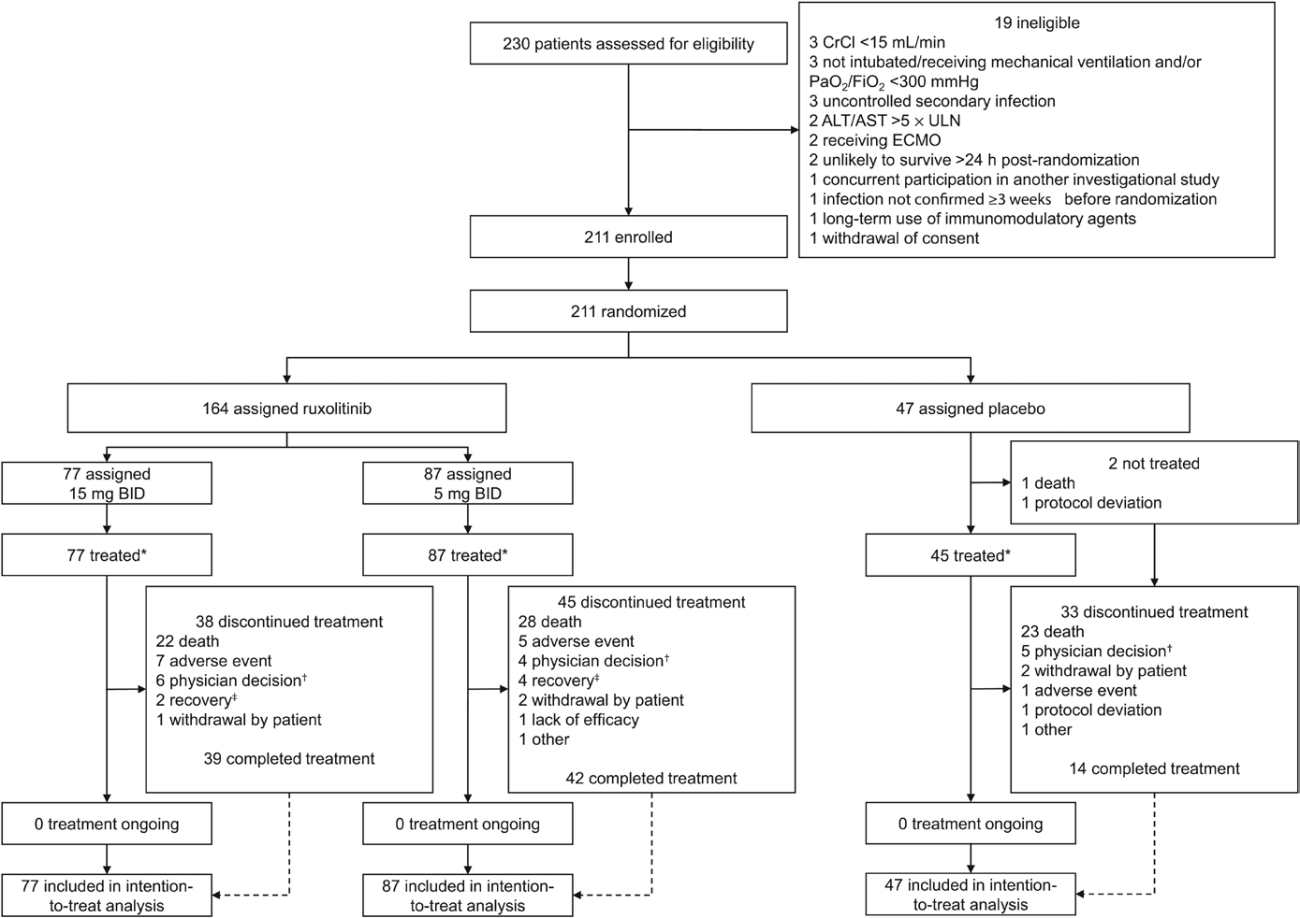
Trial profile. Efficacy analyses were conducted in the intention-to-treat population. *Patient numbers shown are for initial treatment; 73 patients entered the optional second 14-d treatment period (ruxolitinib 15 mg, *n* = 28; ruxolitinib 5 mg, *n* = 35; placebo, *n* = 10). †Reasons for physician decision included increasing creatinine levels (*n* = 1 [ruxolitinib 15 mg]), disease progression (*n* = 2 [ruxolitinib 15 mg, *n* = 1; placebo, *n* = 1]), and other reasons associated with worsening clinical status (*n* = 12 [ruxolitinib 15 mg, *n* = 4; ruxolitinib 5 mg, *n* = 4; placebo, *n* = 4]). ^‡^Patient no longer required treatment. ALT = alanine aminotransferase‚ AST = aspartate aminotransferase‚ CrCl = creatinine clearance‚ ECMO = extracorporeal membrane oxygenation‚ ULN = upper limit of normal.

Between May 24, 2020, and December 15, 2020, 211 patients underwent randomization and were included in the intention-to-treat population (ruxolitinib 15 mg, *n* = 77; ruxolitinib 5 mg, *n* = 87; placebo, *n* = 47) (**Fig. 1**). All but two patients (both in the placebo group) received at least one dose of blinded study treatment and were included in the safety population. Among all patients, mean (sd) age was 63.4 (12.7) years (all ≥ 18 yr), 65% (137/211) were male, and 71% (149/211) were White (**Table 1**). Nine percent of patients (18/211) were enrolled in Russia and 91% (193/211) in the United States. Patient demographics and baseline clinical characteristics were generally balanced across groups, with a slightly higher proportion of men receiving placebo. Overall, 90% of patients (190/211) received prior or concomitant corticosteroids. Mean (sd) SOFA score at baseline was 9.4 (2.5), and baseline WHO nine-point ordinal scores were 6 and 7 for 48% of patients (101/211) and 52% of patients (110/211), respectively. ARDS severity at randomization was categorized as mild/moderate in 73% of patients (153/211) and as severe in 27% of patients (58/211). Comorbid conditions were present in most patients. Median (interquartile range) baseline IL-6 was 27.9 (11.3–114.1) pg/mL (**eTable 2**, http://links.lww.com/CCM/H210).

**TABLE 1.** Patient Demographics and Baseline Clinical Characteristics of the Intention-to-Treat Population

Characteristics	Ruxolitinib 15 mg BID (*n* = 77)	Ruxolitinib 5 mg BID (*n* = 87)	Pooled Ruxolitinib (*n* = 164)	Placebo (*n* = 47)	Total (*n* = 211)
Age, mean (sd), yr	63.6 (12.9)	63.6 (12.3)	63.6 (12.5)	62.5 (13.3)	63.4 (12.7)
Male, *n* (%)	48 (62)	55 (63)	103 (63)	34 (72)	137 (65)
Race, *n* (%)					
White	57 (74)	61 (70)	118 (72)	31 (66)	149 (71)
Black	9 (12)	12 (14)	21 (13)	5 (11)	26 (12)
Other/unknown	11 (14)	14 (16)	25 (15)	11 (23)	36 (17)
Body mass index, mean (sd), kg/m^2^	34.8 (9.8)	33.5 (7.2)	34.1 (8.5)	33.7 (7.4)	34.0 (8.3)
Country, *n* (%)					
United States	71 (92)	78 (90)	149 (91)	44 (94)	193 (91)
Russia	6 (8)	9 (10)	15 (9)	3 (6)	18 (9)
Predefined comorbidities,^a^ *n* (%)	66 (86)	76 (87)	142 (87)	36 (77)	178 (84)
Hypertension	56 (73)	63 (72)	119 (73)	24 (51)	143 (68)
Diabetes	40 (52)	43 (49)	83 (51)	23 (49)	106 (50)
Chronic obstructive pulmonary disease	15 (19)	14 (16)	29 (18)	4 (9)	33 (16)
Chronic kidney disease	7 (9)	14 (16)	21 (13)	7 (15)	28 (13)
Chronic heart disease	10 (13)	10 (11)	20 (12)	6 (13)	26 (12)
Asthma	4 (5)	15 (17)	19 (12)	4 (9)	23 (11)
Sequential Organ Failure Assessment score, mean (sd)	9.2 (2.4)	9.6 (2.5)	9.4 (2.5)	9.4 (2.7)	9.4 (2.5)
COVID-19 9-point ordinal scale, *n* (%)^b^					
6	37 (48)	41 (47)	78 (48)	23 (49)	101 (48)
7	40 (52)	46 (53)	86 (52)	24 (51)	110 (52)
ARDS severity at baseline, *n* (%)					
Pao_2_/Fio_2_ >100–300 mm Hg	59 (77)	66 (76)	125 (76)	33 (70)	158 (75)
Pao_2_/Fio_2_ ≤100 mm Hg	18 (23)	20 (23)	38 (23)	14 (30)	52 (25)
Missing	0	1 (1)	1 (1)	0	1 (<1)
ARDS severity at randomization, *n* (%)					
Pao_2_/Fio_2_ >100–300 mm Hg	58 (75)	66 (76)	124 (76)	29 (62)	153 (73)
Pao_2_/Fio_2_ ≤100 mm Hg	19 (25)	21 (24)	40 (24)	18 (38)	58 (27)
Time from initial diagnosis to randomization, median (IQR), d	9.5 (5.0–15.0)	10.0 (6.0–14.0)	10.0 (5.0–15.0)	9.0 (4.0–15.0)	10.0 (5.0–15.0)
Time from start of mechanical ventilation to randomization, median (IQR), hr	64.2 (37.9–104.5)	57.0 (32.3–144.4)	61.0 (34.6–120.3)	65.0 (34.8–168.0)	61.7 (34.7–121.4)
≤ 48, *n* (%)	29 (38)	35 (40)	64 (39)	17 (36)	81 (38)
> 48, *n* (%)	48 (62)	52 (60)	100 (61)	30 (64)	130 (62)
Prior or concomitant medications, *n* (%)					
Concomitant anticoagulant	72 (94)	82 (94)	154 (94)	42 (89)	196 (93)
Corticosteroid	67 (87)	79 (91)	146 (89)	44 (94)	190 (90)
Remdesivir	42 (55)	44 (51)	86 (52)	29 (62)	115 (55)
Corticosteroid + remdesivir	37 (48)	42 (48)	79 (48)	28 (60)	107 (51)
Convalescent plasma	11 (14)	16 (18)	27 (16)	10 (21)	37 (18)
Prior biologic	3 (4)	8 (9)	11 (7)	0	11 (5)

ARDS = acute respiratory distress syndrome‚ BID = twice a day‚ IQR = interquartile range.

a Reported in > 10% of overall population.

b Scale ranges from 0 to 8; score of 6 defined as intubation and mechanical ventilation and 7 as ventilation plus additional organ support (vasopressors, renal replacement therapy, or extracorporeal membrane oxygenation).

Forty-five percent of patients (95/211) completed the entire 14-day protocol-defined ruxolitinib/placebo course (ruxolitinib 15 mg, 51% [39/77]; ruxolitinib 5 mg, 48% [42/87]; placebo, 30% [14/47]), and 73 patients entered the optional second 14-day treatment period (ruxolitinib 15 mg, *n* = 28; ruxolitinib 5 mg, *n* = 35; placebo, *n* = 10) (Fig. 1). The most common reasons for treatment discontinuation were death (ruxolitinib 15 mg, 29% [22/77]; ruxolitinib 5 mg, 32% [28/87]; placebo, 49% [23/47]), physician decision (ruxolitinib 15 mg, 8% [6/77]; ruxolitinib 5 mg, 5% [4/87]; placebo, 11% [5/47]), and AEs (ruxolitinib 15 mg, 9% [7/77]; ruxolitinib 5 mg, 6% [5/87]; placebo, 2% [1/47]).

The 28-day mortality rate was 51% (39/77; 95% CI, 39–62%) for ruxolitinib 15 mg and 53% (45/85; 95% CI, 42–64%) for ruxolitinib 5 mg versus 70% (33/47; 95% CI, 55–83%) for placebo (**Table 2**). The odds ratios (ORs) were 0.46 (95% CI, 0.201–1.028; one-sided *p* = 0.029) and 0.42 (95% CI, 0.171–1.023; one-sided *p* = 0.028) for 15 mg and 5 mg, respectively, versus placebo. Results of the sensitivity analysis are shown in **eTable 3** (http://links.lww.com/CCM/H210). Subgroup analyses of 28-day mortality demonstrated consistent trends across patient demographic and clinical characteristic subgroups (**eFig. 2**, http://links.lww.com/CCM/H210). Eight of 11 patients (all ruxolitinib-treated) who received prior biologics (all anti–IL-6 antibodies) died by day 29. In a post hoc analysis of the primary endpoint evaluating the U.S. population only (*n* = 191), the 28-day mortality rate was 46.5% (33/71; 95% CI, 34.5%–58.7%) for the ruxolitinib 15 mg treatment group and 47.4% (36/76; 95% CI, 35.8%–59.2%) for ruxolitinib 5 mg versus 68.2% (30/44; 95% CI, 52.4%–81.4%) for placebo (**eTable 4**, http://links.lww.com/CCM/H210). The ORs were 0.43 (95% CI, 0.188–0.974; one-sided *p* = 0.022) and 0.39 (95% CI, 0.157–0.948; one-sided *p* = 0.019) for ruxolitinib 15 mg and 5 mg, respectively, versus placebo. Further, post hoc analysis of the primary outcome pooling the 5 mg and 15 mg ruxolitinib doses for the overall population demonstrated a 28-day mortality rate of 51.9% (84/162; 95% CI, 43.9%–59.8%) with ruxolitinib versus 70.2% (33/47; 95% CI, 55.1%–82.7%) for placebo (OR, 0.47 [95% CI, 0.219–0.996]; one-sided *p* = 0.024) (eTable 4, http://links.lww.com/CCM/H210).

**TABLE 2.** Primary and Secondary Efficacy Outcomes (Intention-to-Treat Population)

Outcome	Ruxolitinib 15 mg BID (*n* = 77)		Ruxolitinib 5 mg BID (*n* = 87)		Placebo (*n* = 47)
Primary outcome					
28-d mortality					
Death due to any cause prior to or on Day 29, *n*/*N* (%)	39/77 (51)		45/85^a^ (53)		33/47 (70)
95% CI for 28-d mortality rate	39.0–62.2		41.8–63.9		55.1–82.7
Odds ratio (95% CI) for ruxolitinib vs placebo	0.46 (0.201–1.028)		0.42 (0.171–1.203)		NA
One-sided *p*	0.029		0.028		NA
Secondary outcomes					
In-hospital outcomes	Mean (sd)	*P * ^b^	Mean (sd)	*P * ^b^	Mean (sd)
Ventilator-free days	6.3 (9.0)	0.015	4.9 (8.4)	0.131	3.0 (7.2)
ICU-free days	4.8 (7.7)	0.017	4.0 (7.5)	0.107	2.5 (6.4)
Vasopressor-free days	9.0 (11.7)	0.014	7.5 (10.9)	0.051	4.5 (9.3)
Hospital-free days	2.1 (4.9)	0.190	2.4 (5.3)	0.155	1.4 (4.0)
Oxygen-free days	2.7 (6.1)	0.201	3.0 (6.7)	0.212	1.5 (4.7)
COVID-19 nine-point ordinal scale score					
Time to any improvement, median (95% CI)	9.0 (7.0–14.0)		13.0 (8.0–19.0)		11.0 (8.0–not evaluable)
Day 15, *n* (%)					
0–2 (uninfected/ambulatory)	4 (5)		5 (6)		2 (4)
3–5 (hospitalized, not intubated)	18 (23)		13 (15)		3 (6)
6–7 (intubated)	31 (40)		42 (48)		13 (28)
8 (dead)	24 (31)		25 (29)		29 (62)
≥ 1-point improvement from baseline, *n*/*N* (%)	32/77 (42)		28/85 (33)		10/47 (21)
Odds ratio (95% CI) for ruxolitinib vs placebo	2.54 (1.067–6.034)		1.72 (0.732–4.041)		NA
*P *^c^	0.0351		0.213		NA
≥ 2-point improvement from baseline, *n*/*N* (%)	17/77 (22)		13/85 (15)		5/47 (11)
Odds ratio (95% CI) for ruxolitinib vs placebo	2.15 (0.724–6.358)		1.32 (0.431–4.036)		NA
*P*^ c^	0.169		0.628		NA
Change from day 1 to day 15, mean (sd)	–0.4 (1.8)		–0.2 (1.7)		0.6 (1.7)
Day 29, *n* (%)					
0–2 (uninfected/ambulatory)	15 (19)		18 (21)		6 (13)
3–5 (hospitalized, not intubated)	10 (13)		6 (7)		2 (4)
6–7 (intubated)	10 (13)		14 (16)		6 (13)
8 (dead)	39 (51)		45 (52)		33 (70)
≥ 1-point improvement from baseline, *n*/*N* (%)	32/74 (43)		27/83 (33)		8/47 (17)
Odds ratio (95% CI) for ruxolitinib vs placebo	3.48 (1.406–8.624)		2.28 (0.899–5.789)		NA
*P *^c^	0.0070		0.0824		NA
≥ 2-point improvement from baseline, *n*/*N* (%)	22/74 (30)		21/83 (25)		8/47 (17)
Odds ratio (95% CI) for ruxolitinib vs placebo	1.89 (0.751–4.759)		1.58 (0.616–4.035)		NA
*P *^c^	0.177		0.342		NA
Change from day 1 to day 29, mean (sd)	–0.5 (2.5)		–0.4 (2.6)		0.4 (2.2)

BID = twice a day, NA = not applicable.

^a^Two patients in the ruxolitinib 5 mg BID group were not evaluable for analysis of the primary endpoint (withdrawn consent).

b Per Kruskal-Wallis test; tested (vs placebo) at the 0.05 level using a two-sided test with no type 1 error allocated.

c Per Wald test from a logistic regression model that included treatment group and acute respiratory distress syndrome severity as fixed effects and investigational site as a random effect; tested (vs placebo) at the 0.05 level using a two-sided test with no type 1 error allocated.

**Figure 2.**
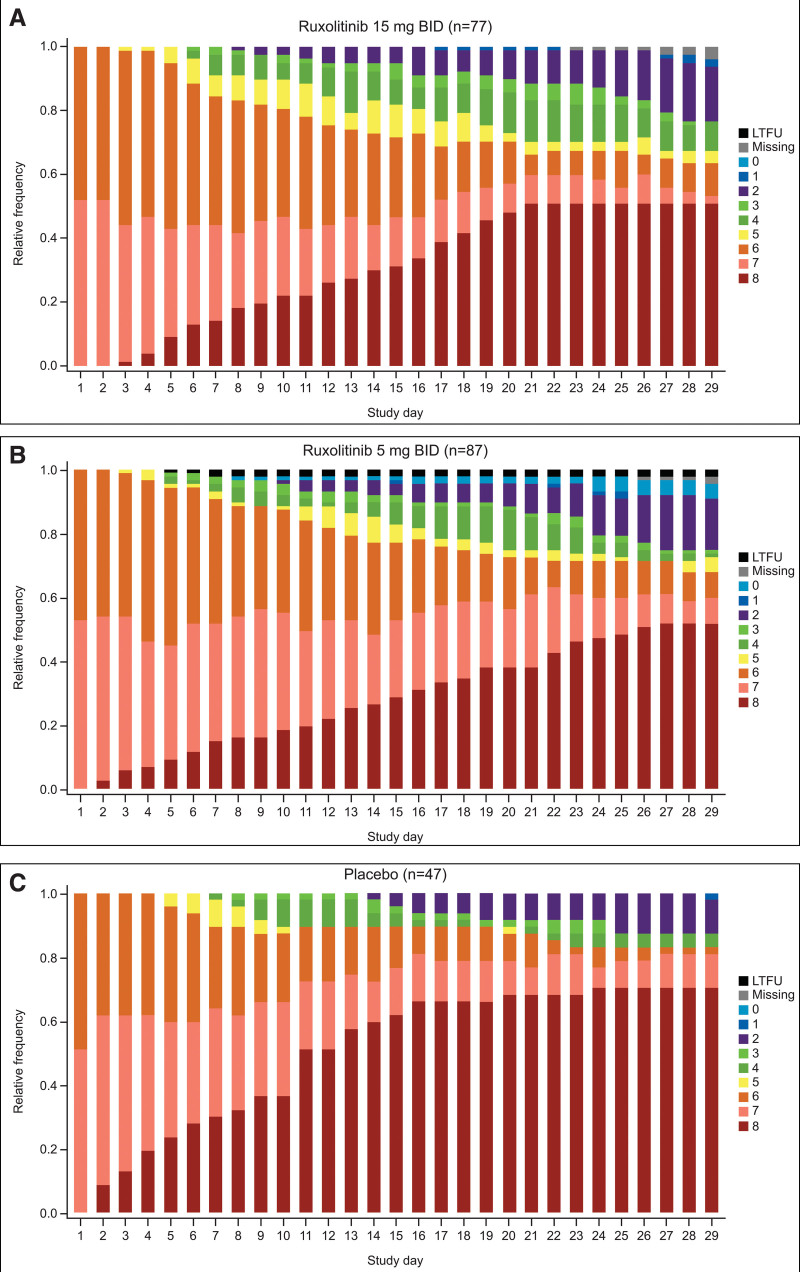
COVID-19 nine-point ordinal scale score by day. **A**, Ruxolitinib 15 mg twice a day (BID), **B**, ruxolitinib 5 mg BID, or **C**, placebo (intention-to-treat population). LTFU = lost to follow-up.

Table 2 shows secondary efficacy outcomes. Patients receiving 15 mg ruxolitinib showed numerical improvements for in-hospital outcomes, including number of ventilator-free, ICU-free, and vasopressor-free days, versus placebo. Hospitalization outcomes for the U.S. population only are presented in **eTable 5** (http://links.lww.com/CCM/H210). Patients treated with ruxolitinib showed a numerical improvement from baseline on the nine-point ordinal scale at days 15 and 29 versus placebo. Ordinal scale scores by trial day (days 1–29) for each treatment group are shown in **Figure 2**. SOFA scores tended to increase (indicating clinical decline) more gradually with ruxolitinib compared with placebo (**eFigs. 3** and **4**, http://links.lww.com/CCM/H210).

Overall rates of TEAEs were similar across trial groups (**Table 3**). The most common TEAEs among patients treated with ruxolitinib (15 mg/5 mg; vs placebo) were anemia (17% [13/77]/24% [21/87] vs 22% [10/45]) and pneumonia (17% [13/77]/16% [14/87] vs 20% [9/45]). The most common grade 3 or higher TEAEs with ruxolitinib versus placebo were pneumonia (16% [12/77]/13% [11/87] vs 18% [8/45]) and anemia (12% [9/77]/14% [12/87] vs 18% [8/45]) (**eTable 6**, http://links.lww.com/CCM/H210). Treatment-related TEAEs are shown in **eTable 7** (http://links.lww.com/CCM/H210). Rates of infections were slightly higher with ruxolitinib versus placebo (any grade, 36% [28/77]/36% [31/87] vs 31% [14/45]; grade ≥ 3, 30% [23/77]/22% [19/87] vs 20% [9/45]) (**eTable 8**, http://links.lww.com/CCM/H210). Serious TEAEs were observed in 30% (23/77)/24% of patients (21/87) and 24% of patients (11/45) treated with ruxolitinib 15 mg/5 mg and placebo, respectively. The most common serious TEAEs with ruxolitinib versus placebo were pneumonia (4% [3/77]/6% [5/87] vs 9% [4/45]) and alanine aminotransferase increased (4% [3/77]/1% [1/87] vs 2% [1/45]) (Table 3). Fatal TEAEs occurred in 6% of patients (10/164) treated with ruxolitinib (*n* = 5/dose) and 11% of patients (5/45) treated with placebo. Fatal TEAEs reported in more than one patient included pneumonia (ruxolitinib 15 mg, *n* = 1; placebo, *n* = 2), cardiopulmonary failure (ruxolitinib 15 mg/5 mg, *n* = 1/1; placebo, *n* = 1), and cerebral hemorrhage (ruxolitinib 5 mg, *n* = 2) (**eTable 9**, http://links.lww.com/CCM/H210). Overall, TEAEs led to dose reductions in 7% of patients (14/209; ruxolitinib 15 mg, 9% [7/77]; ruxolitinib 5 mg, 6% [5/87]; placebo, 4% [2/45]), dose interruptions in 3% of patients (6/209; ruxolitinib 15 mg, 3% [2/77]; ruxolitinib 5 mg, 5% [4/87]; placebo, 0%) and treatment discontinuations in 6% of patients (13/209; ruxolitinib 15 mg, 9% [7/77]; ruxolitinib 5 mg, 6% [5/87]; placebo, 2% [1/45]) (**eTable 10**, http://links.lww.com/CCM/H210).

**TABLE 3.** Treatment-Emergent and Serious Treatment-Emergent Adverse Events (Safety Population)

Event	Ruxolitinib 15 mg BID (*n* = 77)	Ruxolitinib 5 mg BID (*n* = 87)	Placebo (*n* = 45)
Any treatment-emergent adverse event^a^, *n* (%)	59 (77)	66 (76)	32 (71)
Anemia	13 (17)	21 (24)	10 (22)
Pneumonia	13 (17)	14 (16)	9 (20)
Alanine aminotransferase increased	12 (16)	12 (14)	6 (13)
Aspartate aminotransferase increased	12 (16)	11 (13)	4 (9)
Hypertension	7 (9)	12 (14)	5 (11)
Hypokalemia	9 (12)	9 (10)	4 (9)
Hypernatremia	8 (10)	7 (8)	7 (16)
Hypophosphatemia	5 (6)	6 (7)	2 (4)
Hypotension	5 (6)	5 (6)	3 (7)
Hyperglycemia	3 (4)	8 (9)	1 (2)
Hypoalbuminemia	1 (1)	7 (8)	4 (9)
Anxiety	6 (8)	4 (5)	1 (2)
Constipation	5 (6)	4 (5)	2 (4)
Skin ulcer	3 (4)	5 (6)	3 (7)
Pyrexia	2 (3)	5 (6)	4 (9)
Any serious treatment-emergent adverse event,^b^ *n* (%)	23 (30)	21 (24)	11 (24)
Pneumonia	3 (4)	5 (6)	4 (9)
Pneumonia, pathogen unspecified	2 (3)	4 (5)	2 (4)
Pneumonia staphylococcal	1 (1)	1 (1)	0
Pneumonia bacterial	0	0	1 (2)
Pneumonia pseudomonal	0	0	1 (2)
Alanine aminotransferase increased	3 (4)	1 (1)	1 (2)
Hypotension	1 (1)	3 (3)	1 (2)
Hypoxia	1 (1)	1 (1)	3 (7)
Sepsis	3 (4)	1 (1)	0
Pneumothorax	3 (4)	0	1 (2)
Aspartate aminotransferase increased	2 (3)	1 (1)	1 (2)
Hypertension	2 (3)	1 (1)	0
Acute kidney injury	1 (1)	2 (2)	0
Cardiopulmonary failure	1 (1)	1 (1)	1 (2)
Vascular device infection	1 (1)	1 (1)	0
Anemia	1 (1)	0	1 (2)
Pulseless electrical activity	1 (1)	0	1 (2)
Blood alkaline phosphatase increased	0	2 (2)	0
Cerebral hemorrhage	0	2 (2)	0
Lower gastrointestinal hemorrhage	0	2 (2)	0
Peripheral ischemia	0	2 (2)	0
Rectal hemorrhage	0	1 (1)	1 (2)

BID = twice a day.

^a^Treatment-emergent adverse events reported in ≥ 5% of the total patient population are shown.

b Events reported in > 1 patient overall are shown.

## DISCUSSION

Although a numerical reduction in 28-day mortality was observed with ruxolitinib versus placebo (~50% vs 70%, respectively), the results did not reach the prespecified significance level. The 15-mg ruxolitinib regimen also resulted in numerical improvements in several secondary outcomes, including ventilator-free, ICU-free, and vasopressor-free days, COVID-19 WHO ordinal scale score, and SOFA score. Safety findings were consistent with expectations for patients with COVID-19–associated ARDS and ruxolitinib, and no new safety concerns were identified.

Experimental treatments targeting cytokine signaling may mitigate the widespread inflammatory response that leads to lung injury and respiratory failure in severe COVID-19 (4, 5, 13, 27). Several JAK/STAT-regulated cytokines are overexpressed in COVID-19–associated ARDS, and some are predictive of adverse outcomes (8, 13). In the adaptive platform Randomised‚ Embedded‚ Multi-factorial‚ Adaptive Platform Trial for Community-Acquired Pneumonia (REMAP-CAP) trial, treatment of hospitalized patients with the IL-6 receptor antagonists tocilizumab or sarilumab improved outcomes, including survival, compared with usual care alone (10). Accordingly, tocilizumab (plus dexamethasone) was added to National Institutes of Health guidelines as a treatment option for COVID-19 with rapid respiratory decompensation (28) and is authorized for emergency use in the United States. However, clinical trials have yielded conflicting results on the benefit of tocilizumab. Separate phase 3 studies showed no benefit of tocilizumab versus placebo in moderately ill patients (29) and no improvement in clinical status at day 28 or survival in another study of hospitalized patients with severe COVID-19 (17). Furthermore, less than 15% of patients in the open-label Randomised Evaluation of COVID-19 Therapy (RECOVERY) platform trial were mechanically ventilated, with similar mortality rates for tocilizumab and usual care among these patients (30). It is therefore reasonable to target multiple inflammatory cytokines in addition to IL-6 via inhibition of the JAK/STAT pathway (27).

Proof of concept was demonstrated with the JAK1/JAK2 inhibitor baricitinib, which improved recovery time among patients with COVID-19–associated pneumonia when added to remdesivir (22). Baricitinib later improved 28-day mortality among hospitalized patients versus standard of care in the Baricitinib in Participants With COVID-19 (COV-BARRIER) study (23), including among critically ill patients receiving mechanical ventilation (11). Of note, COV-BARRIER did not meet its primary composite endpoint (progression to oxygen/ventilation requirement or death by day 28). A small, prospective, open-label, nonrandomized phase 2 study of patients with ARDS reported clinical benefit of ruxolitinib, including a 28-day survival rate of 81% (13/16 patients) (21). In contrast, the phase 3 placebo-controlled Ruxolitinib in Patients With COVID-19–Associated Cytokine Storm study (RUXCOVID) of ruxolitinib versus placebo in nonmechanically ventilated hospitalized patients did not reduce mortality, progression to respiratory failure, or need for ICU care by day 29 (31). The current trial builds upon previous experience with ruxolitinib by contributing data on treatment outcomes in patients with COVID-19–associated ARDS in a randomized, placebo-controlled clinical setting. Taken together with previous reports, these findings suggest that ruxolitinib may have added benefits in patients who are critically ill from COVID-19–associated ARDS, even those on corticosteroids.

Several limitations should be considered. The trial was terminated before target enrollment was reached, and the final sample size was less than half of that deemed necessary to detect significant differences between treatment groups. Further, the 2:2:1 randomization schema, which was employed to maximize the number of patients receiving ruxolitinib in light of the critical unmet need for alternative therapies at the time the trial was initiated, was not fully balanced at the time of early termination, with fewer patients receiving ruxolitinib at final enrollment than would be expected based on the randomization schema. These complications reflect the larger COVID-19 clinical trial landscape, in which the rapidly evolving nature of the pandemic and the substantial time and resource demands to implement properly conducted trials have led to an inundation of underpowered studies yielding difficult-to-interpret findings. A global assessment of clinical development efforts reported that approximately 5% of all trial arms in COVID-19 studies ultimately met criteria for randomization and adequate power, with only 26% of all trial participants contributing to adequately powered, well-controlled studies (32).

In addition to the noted limitations related to early termination, higher proportions of patients receiving placebo versus ruxolitinib received prior or concomitant corticosteroids or remdesivir. Additionally, no patients randomized to placebo had received prior biologics compared with 11 ruxolitinib-treated patients (7%) (all anti–IL-6 antibodies), eight of whom died during the study. The study allowed enrollment up to 3 weeks from SARS-CoV-2 infection, and patients may have been in different phases of ARDS at the time of randomization and throughout the course of the study. Patients were not stratified by ARDS phase (i.e., early, mid, late) at baseline, which could have impacted outcomes, as response to treatment and overall prognosis may vary according to ARDS phase (33). Furthermore, observed mortality rates were slightly higher than expected (based on limited mortality rate estimates) for both the ruxolitinib and placebo groups (approximately 50% and 70% vs projections of 40% and 60%, respectively). The bulk of trial enrollment occurred during the peak of the pandemic in the United States, placing significant pressure on ICU staffing and causing a relative shortage of standard resources, which may have contributed to higher than anticipated mortality at some trial sites (34). Additional contributing factors include the changing standard of care toward treating patients with less severe disease with noninvasive positive pressure ventilation and high-flow nasal cannula for extended periods of time, resulting in more severe lung disease at intubation in those failing initial therapy, high rates of comorbidities, and 100% mortality in Russia (all cohorts).

## CONCLUSIONS

This trial did not reach the prespecified significance level for its primary endpoint. However, ruxolitinib treatment yielded some encouraging results among patients with COVID-19–associated ARDS, including numerically improved in-hospital outcomes and clinical status. As the study was terminated early and ultimately underpowered to assess efficacy outcomes, additional evaluations are required to determine whether ruxolitinib improves outcomes for critically ill patients.

## ACKNOWLEDGMENTS

Writing assistance was provided by Jane Kovalevich, PhD, an employee of ICON (Blue Bell, PA), and was funded by Incyte Corporation (Wilmington, DE).

## Supplementary Material


