# Supplementary material for: Randomized Phase 3 Trial of Ruxolitinib for COVID-19–Associated Acute Respiratory Distress Syndrome*
Source: Crit Care Med. 2022 Oct 13;50(12):1701–13. doi: 10.1097/CCM.0000000000005682 (PMC9668361; doi:10.1097/CCM.0000000000005682)
Supplement: Supplementary file 1 [file ccm-50-1701-s001.docx]

**Randomized Phase 3 Trial of Ruxolitinib for Coronavirus Disease 2019–Associated Acute Respiratory Distress Syndrome**

Lindsay Rein, MD, Karel Calero, MD, Ronak Shah, MD, Charles Ojielo, MD, Kristin M. Hudock, MD, MSTR, Saba Lodhi, MD, Farid Sadaka, MD, Shashi Bellam, MD, Christopher Palma, MD, ScM, David N. Hager, MD, PhD, Jeannie Daniel, PhD, Richard Schaub, MS, Kevin O’Hayer, MD, PhD, Nicole M. Theodoropoulos, MD, MS

**Supplemental Digital Content**

[Supplementary Methods 36](#_Toc102733863)

[Supplementary Figures 37](#_Toc102733864)

[eFigure 1. RUXCOVID-DEVENT Trial Design 37](#_Toc102733865)

[eFigure 2. Subgroup Analysis of the Primary Endpoint 38](#_Toc102733866)

[eFigure 3. Mean Change From Baseline in SOFA Score Over Time 40](#_Toc102733867)

[eFigure 4. SOFA Score by Treatment Group. SOFA score by study day for (A) all evaluable patients and (B) surviving patients only 41](#_Toc102733868)

[Supplementary Tables 42](#_Toc102733869)

[eTable 1. COVID-19 9-Point Ordinal Clinical Status 42](#_Toc102733870)

[eTable 2. Baseline Laboratory Values 43](#_Toc102733871)

[eTable 3. Preplanned Sensitivity Analysis of the Primary Outcome^a^ 44](#_Toc102733872)

[eTable 4. Post Hoc Analyses of the Primary Outcome 45](#_Toc102733873)

[eTable 5. In-Hospital Outcomes Among the US Patient Cohort (Intention-to-Treat Population) 46](#_Toc102733874)

[eTable 6. Grade ≥3 Treatment-Emergent Adverse Events (Safety Population) 47](#_Toc102733875)

[eTable 7. Treatment-Related Adverse Events (Safety Population) 48](#_Toc102733876)

[eTable 8. Treatment-Emergent Infections (Safety Population) 50](#_Toc102733877)

[eTable 9. Treatment-Emergent Adverse Events With a Fatal Outcome (Safety Population) 52](#_Toc102733878)

[eTable 10. Treatment-Emergent Adverse Events Leading to Dose Modifications (Safety Population) 53](#_Toc102733879)

# Supplementary Methods

RUXCOVID-DEVENT was conducted at 29 hospitals and community practices in the United States and 4 hospitals in Russia. Patients were randomized 2:2:1 to ruxolitinib 15 mg twice daily, ruxolitinib 5 mg twice daily, or matching dual-dose placebo. Treatment identity was concealed via use of identical packaging, schedule administration, appearance, taste, and odor. Clinical status using the coronavirus disease 2019 (COVID-19) World Health Organization (WHO) 9-point ordinal scale was evaluated daily from screening through Day 29, as were arterial blood gases, arterial oxygen partial pressure/fractional inspired oxygen (PaO_2_/FiO_2_), pulse oximetry, ventilator/oxygen support, in-hospital outcomes, and sequential organ failure assessment (SOFA) score as possible per clinical status and care environment. SOFA score was determined as previously described (35), with total score based on the sum of 6 different scores. The COVID-19 WHO 9-point ordinal scale was implemented in accordance with published guidelines (eTable 1) (25). AE severity was graded according to Common Terminology Criteria for Adverse Events v5.0.

# Supplementary Figures

## **eFigure 1. RUXCOVID-DEVENT Trial Design**


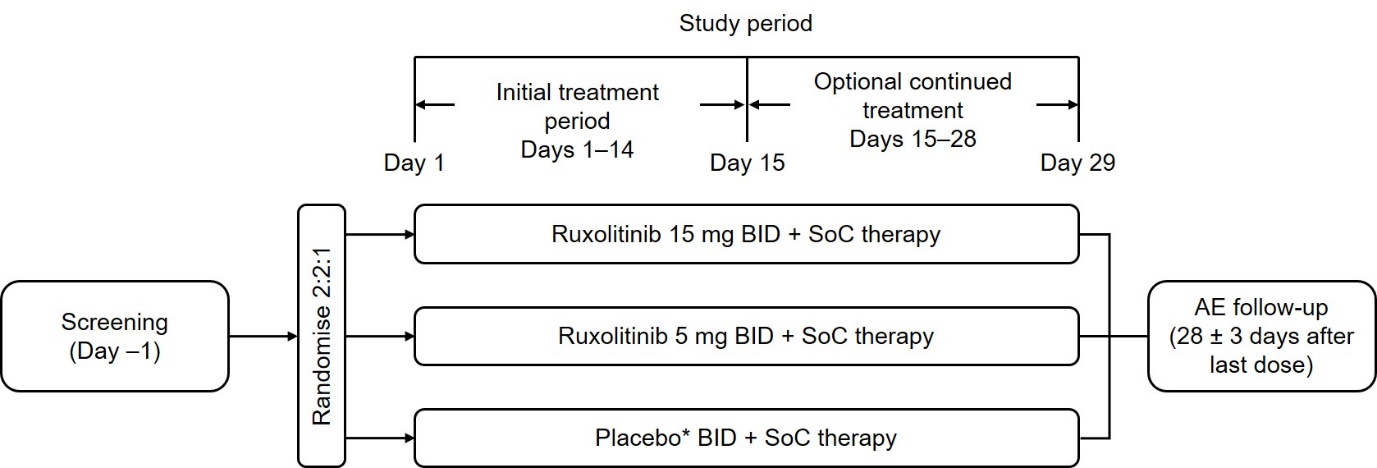


AE=adverse event; BID=twice daily; SoC=standard of care.
* Placebo group was randomized between 5 mg BID (1 tablet/dose) and 15 mg BID (3 tablets/dose).

## **eFigure 2. Subgroup Analysis of the Primary Endpoint**

(A) Ruxolitinib 15 mg BID and (B) ruxolitinib 5 mg BID vs placebo (intention-to-treat population)

**
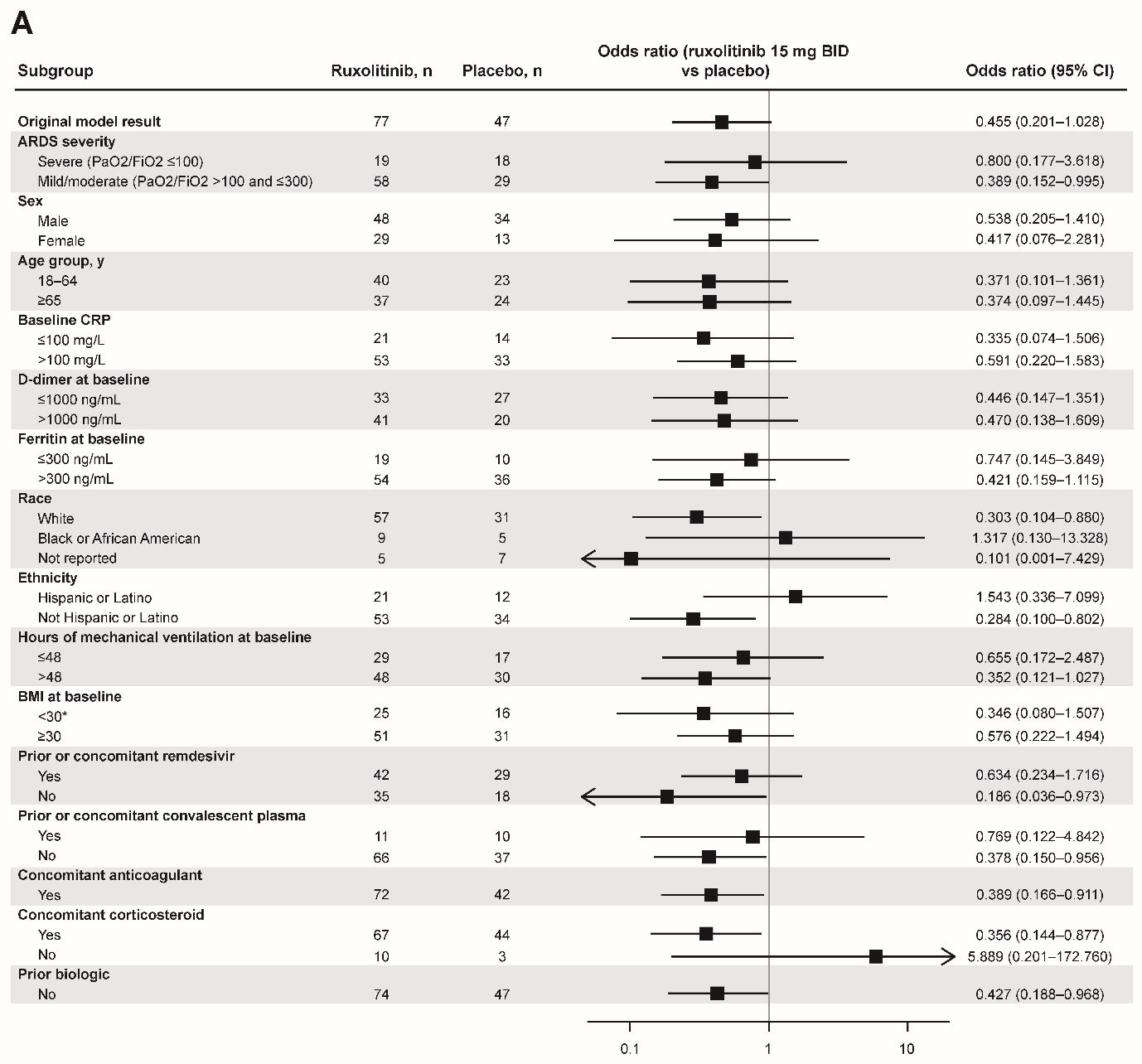
**

**
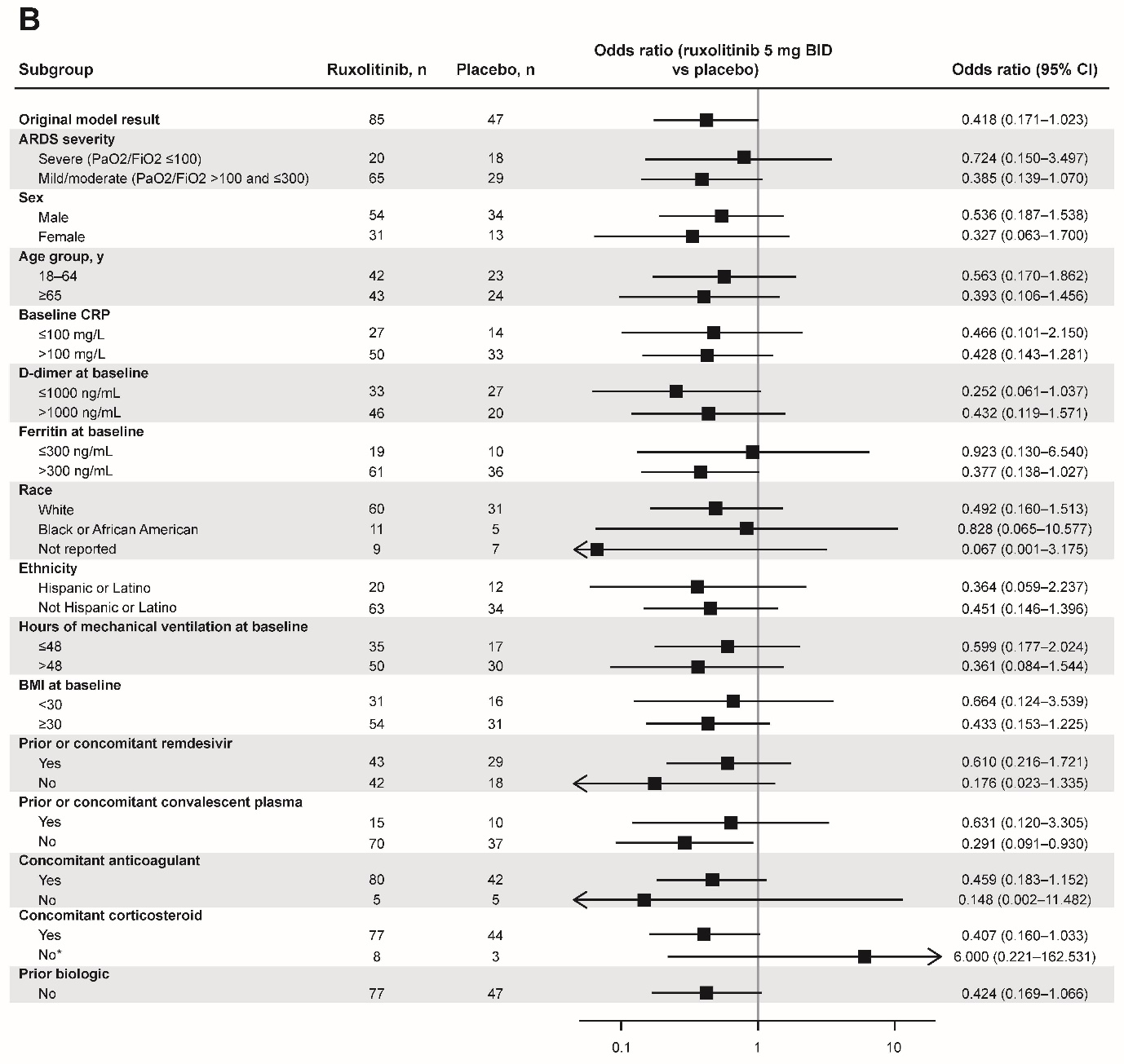
**

ARDS=acute respiratory distress syndrome; BID=twice daily; BMI=body mass index; CRP=C-reactive protein; FiO2=fractional inspired oxygen; PaO2=arterial oxygen partial pressure.
* The mixed effect model did not converge and a fixed effect logistic regression was performed instead, excluding the investigational site from the model.

## **eFigure 3. Mean Change From Baseline in SOFA Score Over Time**

*
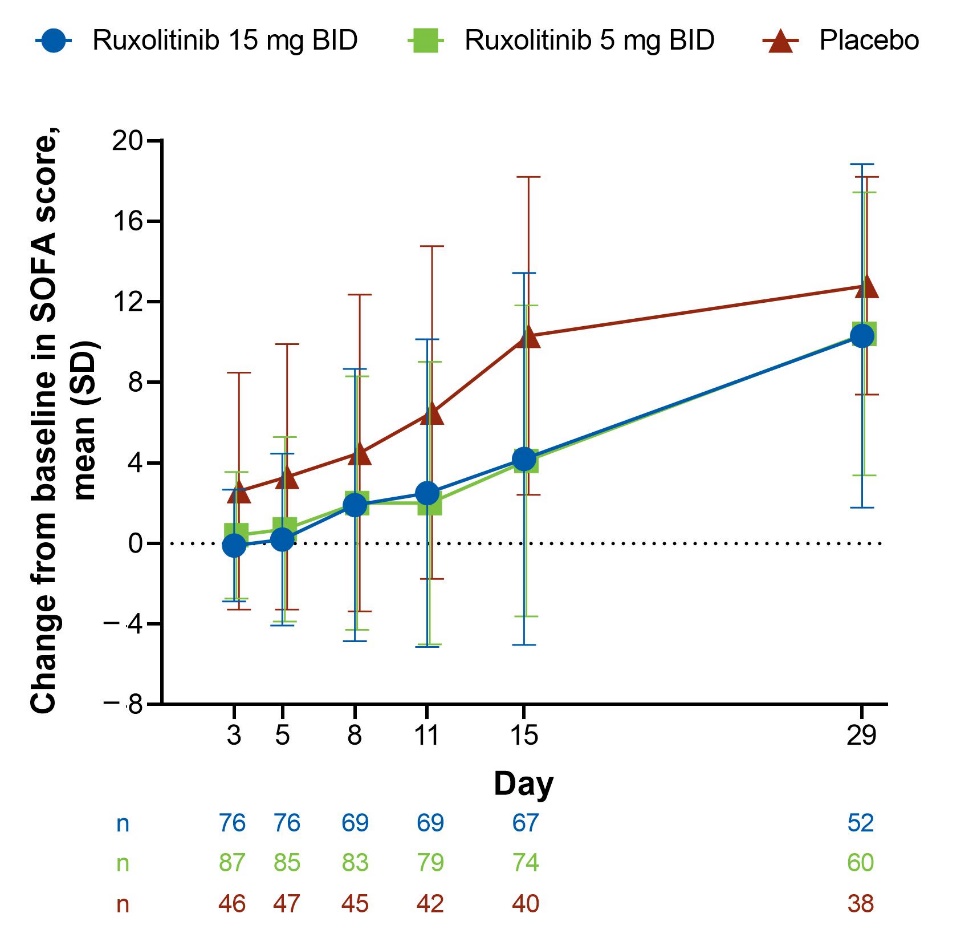
*

BID=twice daily; SOFA=sequential organ failure assessment.

## **eFigure 4. SOFA Score by Treatment Group.** SOFA score by study day for (A) all evaluable patients and (B) surviving patients only


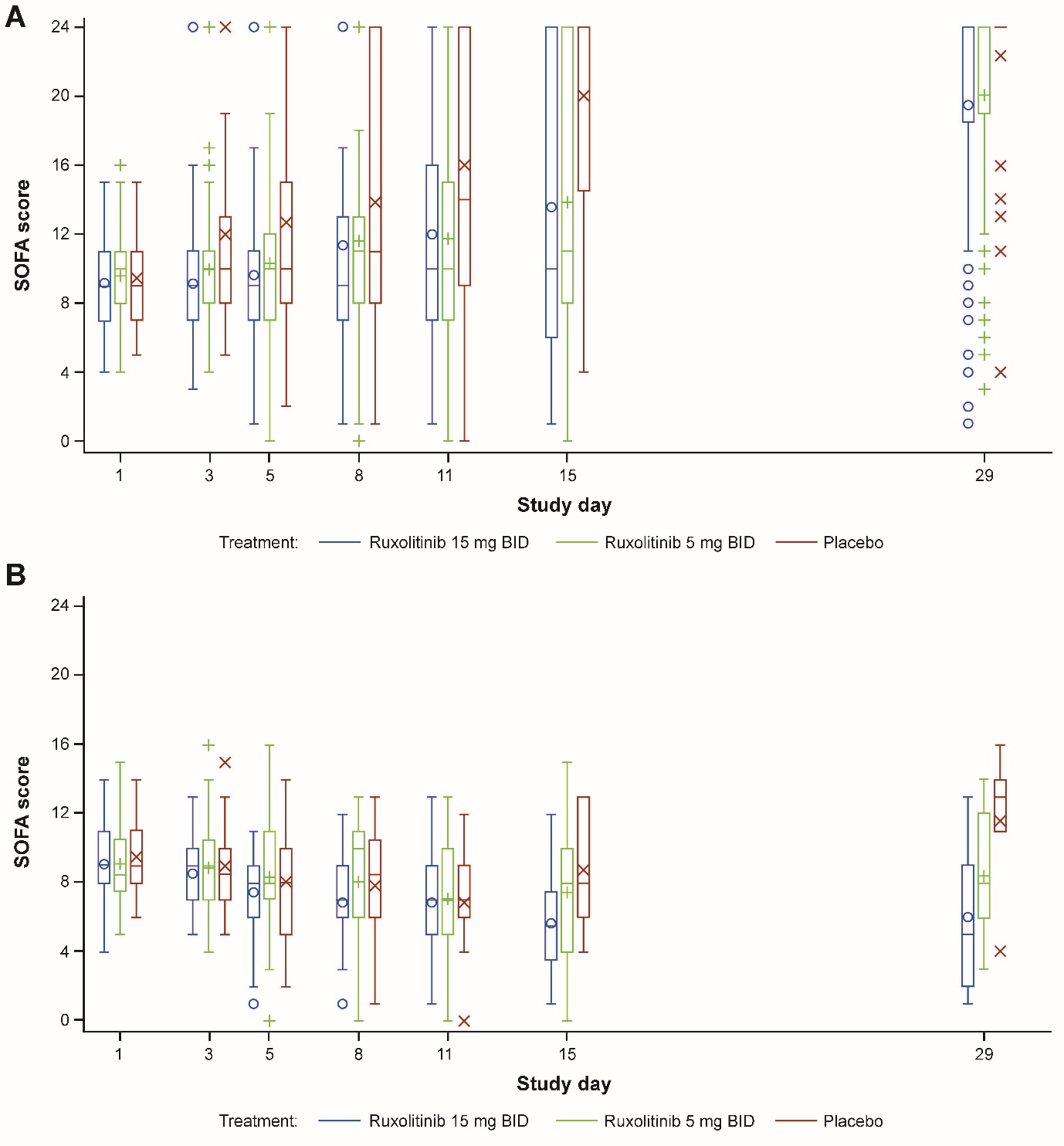


BID=twice daily; SOFA=sequential organ failure assessment.

# Supplementary Tables

## **eTable 1. COVID-19 9-Point Ordinal Clinical Status**

| **Participant State** | **Descriptor** | **Score** |
| --- | --- | --- |
| Uninfected | No clinical or virologic evidence of infection | 0 |
| Ambulatory*^a^* | No limitations of activities | 1 |
|  | Limitation of activities | 2 |
| Hospitalized mild disease | Hospitalized, no oxygen therapy (defined as SpO_2_ ≥94% on room air) | 3 |
|  | Oxygen by mask or nasal prongs | 4 |
| Hospitalized severe disease | Noninvasive ventilation or high-flow oxygen | 5 |
|  | Intubation and mechanical ventilation | 6 |
|  | Ventilation + additional organ support – vasopressors, RRT, ECMO | 7 |
| Dead | Death | 8 |

COVID-19=coronavirus disease 2019; ECMO=extracorporeal membrane oxygenation; RRT=renal replacement therapy.
*^a^* Defined as not in hospital or in hospital and ready for discharge.
Source: World Health Organization: COVID-19 Therapeutic Trial Synopsis.Available at: <https://www.who.int/publications/i/item/covid-19-therapeutic-trial-synopsis>. Accessed March 25, 2021

## **eTable 2. Baseline Laboratory Values**

| **Parameter, median (IQR)** | **Ruxolitinib 15 mg BID (n=77)** | **Ruxolitinib 5 mg BID (n=87)** | **Pooled**  **ruxolitinib (n=164)** | **Placebo (n=47)** | **Total (N=211)** |
| --- | --- | --- | --- | --- | --- |
| CRP, mg/L | 192.5 (89.5–400.0) | 151.6 (72.0–329.8) | 165.5 (78.7–366.0) | 174.5 (68.6–299.9) | 167.7 (75.0–350.9) |
| D-dimer, ng/mL | 1148.5 (20.0–3740.0) | 1169.0 (323.0–3731.0) | 1169.0 (20.0–3740.0) | 703.0 (13.0–2620.0) | 1112.5 (20.0–3305.0) |
| Ferritin, ng/mL | 603.0 (289.3–1063.0) | 650.9 (318.0–1280.0) | 627.5 (293.0–1242.0) | 664.0 (343.0–1459.5) | 644.7 (318.0–1265.1) |
| IL-6, pg/mL | 38.4 (11.9–135.3) | 23.0 (9.3–53.5) | 27.5 (10.6–106.1) | 30.5 (17.5–123.1) | 27.9 (11.3–114.1) |

BID=twice daily; CRP=C-reactive protein; IL=interleukin; IQR=interquartile range.

## **eTable 3. Preplanned Sensitivity Analysis of the Primary Outcome^a^**

| **28-Day mortality** | **Ruxolitinib 15 mg BID (n=77)** | **Ruxolitinib 5 mg BID (n=87)** | **Placebo (n=47)** |
| --- | --- | --- | --- |
| Death due to any cause prior to or on Day 29, n (%) | 39 (50.6) | 47 (54.0) | 33 (70.2) |
| 95% CI for 28-day mortality rate | 39.0–62.2 | 43.0–64.8 | 55.1–82.7 |
| Odds ratio (95% CI) for ruxolitinib vs placebo | 0.46 (0.201–1.028) | 0.42 (0.172–1.039) | – |

BID=twice daily.

***^a^*** A preplanned sensitivity analysis of the primary endpoint was conducted in which patients who were lost to follow-up (n=2; both ruxolitinib 5 mg) were imputed as deaths.

## **eTable 4. Post Hoc Analyses of the Primary Outcome**

| **Outcome** | **Ruxolitinib 15 mg BID** | **Ruxolitinib 5 mg BID*^a^*** | **Placebo** |
| --- | --- | --- | --- |
| 28-Day mortality in the US intention-to-treat population |  |  |  |
| Death due to any cause prior to or on Day 29, n/evaluable n (%) | 33/71 (46.5) | 36/76 (47.4) | 30/44 (68.2) |
| 95% CI for 28-day mortality rate | 34.5–58.7 | 35.8–59.2 | 52.4–81.4 |
| Odds ratio (95% CI) for ruxolitinib vs placebo | 0.43 (0.188–0.974) | 0.39 (0.157–0.948) | – |
| *P* value for ruxolitinib vs placebo | 0.0215 | 0.0189 | – |
| 28-Day mortality in pooled ruxolitinib groups (intention-to-treat population) |  | |  |
| Death due to any cause prior to or on Day 29, n/evaluable n (%) | 84/162 (51.9) | | 33/47 (70.2) |
| 95% CI for 28-day mortality rate | 43.9–59.8 | | 55.1–82.7 |
| Odds ratio (95% CI) for ruxolitinib vs placebo | 0.47 (0.219–0.996) | | – |
| *P* value for ruxolitinib vs placebo | 0.0244 | | – |

BID=twice daily.
*^a^* Two patients in the ruxolitinib 5 mg BID group were not evaluable for analysis of the primary endpoint (withdrawn consent).

## **eTable 5. In-Hospital Outcomes Among the US Patient Cohort (Intention-to-Treat Population)**

| **Outcome** | **Ruxolitinib 15 mg BID**  **(n=71)** | **Ruxolitinib 5 mg BID**  **(n=78)** | **Placebo**  **(n=44)** |
| --- | --- | --- | --- |
| Ventilator-free days | 6.9 (9.2) | 5.5 (8.7) | 3.2 (7.4) |
| *P* value for ruxolitinib vs placebo^a^ | 0.012 | 0.094 | – |
| ICU-free days | 5.2 (7.9) | 4.5 (7.8) | 2.6 (6.5) |
| *P* value for ruxolitinib vs placebo^a^ | 0.014 | 0.080 | – |
| Vasopressor-free days | 9.7 (11.9) | 8.4 (11.2) | 4.8 (9.6) |
| *P* value for ruxolitinib vs placebo^a^ | 0.011 | 0.032 | – |
| Hospital-free days | 2.3 (5.1) | 2.6 (5.5) | 1.5 (4.1) |
| *P* value for ruxolitinib vs placebo^a^ | 0.177 | 0.121 | – |
| Oxygen-free days | 2.9 (6.3) | 3.3 (7.1) | 1.6 (4.8) |
| *P* value for ruxolitinib vs placebo^a^ | 0.185 | 0.169 | – |

Data are mean (SD).
BID=twice daily; ICU=intensive care unit.
^a^ Per Kruskal-Wallis test; tested (vs placebo) at the 0.05 level using a 2-sided test with no type 1 error allocated.

## **eTable 6. Grade ≥3 Treatment-Emergent Adverse Events (Safety Population)**

| **Event** | **Ruxolitinib  15 mg BID (n=77)** | **Ruxolitinib  5 mg BID (n=87)** | **Placebo (n=45)** |
| --- | --- | --- | --- |
| Any grade ≥3 treatment-emergent adverse event,*^a^* n (%) | 42 (54.5) | 47 (54.0) | 25 (55.6) |
| Pneumonia | 12 (15.6) | 11 (12.6) | 8 (17.8) |
| Anemia | 9 (11.7) | 12 (13.8) | 8 (17.8) |
| Hypertension | 6 (7.8) | 11 (12.6) | 3 (6.7) |
| Hypotension | 4 (5.2) | 4 (4.6) | 2 (4.4) |
| Alanine aminotransferase increased | 4 (5.2) | 3 (3.4) | 1 (2.2) |
| Aspartate aminotransferase increased | 4 (5.2) | 3 (3.4) | 1 (2.2) |
| Lymphocyte count decreased | 2 (2.6) | 3 (3.4) | 3 (6.7) |
| Acute kidney injury | 4 (5.2) | 2 (2.3) | 1 (2.2) |
| Sepsis | 3 (3.9) | 2 (2.3) | 0 |
| Hyperglycemia | 2 (2.6) | 3 (3.4) | 0 |
| Acidosis | 2 (2.6) | 2 (2.3) | 1 (2.2) |
| Pneumothorax | 2 (2.6) | 2 (2.3) | 1 (2.2) |
| Atrial fibrillation | 1 (1.3) | 2 (2.3) | 2 (4.4) |
| Hypoxia | 1 (1.3) | 1 (1.1) | 3 (6.7) |
| Hypophosphatemia | 2 (2.6) | 2 (2.3) | 0 |
| Hypokalemia | 1 (1.3) | 2 (2.3) | 1 (2.2) |
| Chronic kidney disease | 1 (1.3) | 1 (1.1) | 2 (4.4) |
| Staphylococcal infection | 2 (2.6) | 1 (1.1) | 0 |
| Urinary tract infection | 2 (2.6) | 1 (1.1) | 0 |
| Blood creatinine increased | 2 (2.6) | 0 | 1 (2.2) |
| Endotracheal intubation complication | 1 (1.3) | 2 (2.3) | 0 |
| Pneumomediastinum | 1 (1.3) | 2 (2.3) | 0 |
| Septic shock | 1 (1.3) | 2 (2.3) | 0 |
| Cardiopulmonary failure | 1 (1.3) | 1 (1.1) | 1 (2.2) |
| Leukocytosis | 1 (1.3) | 1 (1.1) | 1 (2.2) |
| Transaminases increased | 0 | 2 (2.3) | 1 (2.2) |
| Hyperkalemia | 0 | 1 (1.1) | 2 (4.4) |

BID=twice daily.
*^a^* Events reported in >2 patients overall are shown.

## **eTable 7. Treatment-Related Adverse Events (Safety Population)**

| **Event** | **Ruxolitinib**  **15 mg BID (n=77)** | **Ruxolitinib**  **5 mg BID (n=87)** | **Placebo (n=45)** |
| --- | --- | --- | --- |
| Any treatment-related adverse event, n (%) | 22 (28.6) | 23 (26.4) | 11 (24.4) |
| Anemia | 6 (7.8) | 10 (11.5) | 2 (4.4) |
| Aspartate aminotransferase increased | 8 (10.4) | 4 (4.6) | 1 (2.2) |
| Alanine aminotransferase increased | 7 (9.1) | 4 (4.6) | 2 (4.4) |
| Hypertension | 3 (3.9) | 2 (2.3) | 0 |
| Thrombocytopenia | 2 (2.6) | 2 (2.3) | 1 (2.2) |
| Constipation | 2 (2.6) | 2 (2.3) | 0 |
| Pneumonia | 1 (1.3) | 4 (4.6) | 1 (2.2) |
| Pneumonia, pathogen unspecified | 1 (1.3) | 3 (3.4) | 0 |
| Pneumonia staphylococcal | 0 | 1 (1.1) | 0 |
| Pneumonia pseudomonal | 0 | 0 | 1 (2.2) |
| Urinary tract infection | 0 | 2 (2.3) | 1 (2.2) |
| Sepsis | 2 (2.6) | 0 | 0 |
| Staphylococcal infection | 1 (1.3) | 1 (1.1) | 0 |
| Pyrexia | 0 | 1 (1.1) | 1 (2.2) |
| Enterobacter infection | 1 (1.3) | 0 | 0 |
| Fungemia | 1 (1.3) | 0 | 0 |
| Gastroesophageal reflux disease | 1 (1.3) | 0 | 0 |
| Lymphocyte count decreased | 1 (1.3) | 0 | 0 |
| Staphylococcal sepsis | 1 (1.3) | 0 | 0 |
| Strongyloidiasis | 1 (1.3) | 0 | 0 |
| Vomiting | 1 (1.3) | 0 | 0 |
| White blood cell count decreased | 1 (1.3) | 0 | 0 |
| White blood cell count increased | 1 (1.3) | 0 | 0 |
| Bacteremia | 0 | 1 (1.1) | 0 |
| Bandemia | 0 | 1 (1.1) | 0 |
| Blood alkaline phosphatase increased | 0 | 1 (1.1) | 0 |
| Candida infection | 0 | 1 (1.1) | 0 |
| Diarrhea | 0 | 1 (1.1) | 0 |
| Immune reconstitution inflammatory syndrome | 0 | 1 (1.1) | 0 |
| Pseudomonas test positive | 0 | 1 (1.1) | 0 |
| Staphylococcal bacteremia | 0 | 1 (1.1) | 0 |
| Bacterial sepsis | 0 | 0 | 1 (2.2) |
| Hypoxia | 0 | 0 | 1 (2.2) |
| Nausea | 0 | 0 | 1 (2.2) |
| Platelet count decreased | 0 | 0 | 1 (2.2) |
| Streptococcal bacteremia | 0 | 0 | 1 (2.2) |
| Transaminases increased | 0 | 0 | 1 (2.2) |

BID=twice daily.

## **eTable 8. Treatment-Emergent Infections (Safety Population)**

| **Infection** | **Ruxolitinib**  **15 mg BID (n=77)** | **Ruxolitinib**  **5 mg BID (n=87)** | **Placebo (n=45)** |
| --- | --- | --- | --- |
| Any treatment-emergent infection, n (%) | 28 (36.4) | 31 (35.6) | 14 (31.1) |
| Pneumonia | 13 (16.9) | 14 (16.1) | 9 (20.0) |
| Pneumonia, pathogen unspecified | 5 (6.5) | 6 (6.9) | 3 (6.7) |
| Pneumonia staphylococcal | 6 (7.8) | 3 (3.4) | 1 (2.2) |
| Pneumonia klebsiella | 2 (2.6) | 4 (4.6) | 1 (2.2) |
| Pneumonia bacterial | 2 (2.6) | 0 | 3 (6.7) |
| Enterobacter pneumonia | 1 (1.3) | 1 (1.1) | 0 |
| Pneumonia pneumococcal | 0 | 1 (1.1) | 0 |
| Pneumonia pseudomonal | 0 | 0 | 1 (2.2) |
| Urinary tract infection | 3 (3.9) | 3 (3.4) | 1 (2.2) |
| Bacteremia | 1 (1.3) | 5 (5.7) | 0 |
| Sepsis | 3 (3.9) | 2 (2.3) | 0 |
| Staphylococcal infection | 3 (3.9) | 2 (2.3) | 0 |
| Candida infection | 0 | 2 (2.3) | 1 (2.2) |
| Oral candidiasis | 0 | 1 (1.1) | 2 (4.4) |
| Septic shock | 1 (1.3) | 2 (2.3) | 0 |
| Urinary tract infection bacterial | 1 (1.3) | 1 (1.1) | 1 (2.2) |
| Aspergillus infection | 0 | 2 (2.3) | 0 |
| Enterobacter infection | 2 (2.6) | 0 | 0 |
| Lower respiratory tract infection bacterial | 1 (1.3) | 1 (1.1) | 0 |
| Staphylococcal bacteremia | 0 | 2 (2.3) | 0 |
| Stenotrophomonas infection | 2 (2.6) | 0 | 0 |
| Vascular device infection | 1 (1.3) | 1 (1.1) | 0 |
| Bacterial sepsis | 0 | 0 | 1 (2.2) |
| Citrobacter infection | 0 | 1 (1.1) | 0 |
| Fungemia | 1 (1.3) | 0 | 0 |
| Hepatic infection | 0 | 1 (1.1) | 0 |
| Klebsiella infection | 1 (1.3) | 0 | 0 |
| Penile infection | 1 (1.3) | 0 | 0 |
| Pseudomembranous colitis | 0 | 1 (1.1) | 0 |
| Serratia infection | 1 (1.3) | 0 | 0 |
| Sinusitis | 1 (1.3) | 0 | 0 |
| Staphylococcal sepsis | 1 (1.3) | 0 | 0 |
| Stoma site infection | 0 | 1 (1.1) | 0 |
| Streptococcal bacteremia | 0 | 0 | 1 (2.2) |
| Strongyloidiasis | 1 (1.3) | 0 | 0 |
| Systemic candida | 0 | 1 (1.1) | 0 |
| Tracheitis | 0 | 1 (1.1) | 0 |
| Tracheobronchitis bacterial | 0 | 1 (1.1) | 0 |
| Urinary tract infection pseudomonal | 0 | 1 (1.1) | 0 |
| Any grade ≥3 treatment-emergent infection, n (%) | 23 (29.9) | 19 (21.8) | 9 (20.0) |
| Pneumonia | 12 (15.6) | 11 (12.6) | 8 (17.8) |
| Pneumonia, pathogen unspecified | 5 (6.5) | 6 (6.9) | 3 (6.7) |
| Pneumonia staphylococcal | 5 (6.5) | 2 (2.3) | 0 |
| Pneumonia klebsiella | 2 (2.6) | 3 (3.4) | 1 (2.2) |
| Pneumonia bacterial | 2 (2.6) | 0 | 3 (6.7) |
| Enterobacter pneumonia | 1 (1.3) | 0 | 0 |
| Pneumonia pneumococcal | 0 | 1 (1.1) | 0 |
| Pneumonia pseudomonal | 0 | 0 | 1 (2.2) |
| Sepsis | 3 (3.9) | 2 (2.3) | 0 |
| Septic shock | 1 (1.3) | 2 (2.3) | 0 |
| Staphylococcal infection | 2 (2.6) | 1 (1.1) | 0 |
| Urinary tract infection | 2 (2.6) | 1 (1.1) | 0 |
| Bacteremia | 1 (1.3) | 1 (1.1) | 0 |
| Enterobacter infection | 2 (2.6) | 0 | 0 |
| Vascular device infection | 1 (1.3) | 1 (1.1) | 0 |
| Aspergillus infection | 0 | 1 (1.1) | 0 |
| Bacterial sepsis | 0 | 0 | 1 (2.2) |
| Candida infection | 0 | 1 (1.1) | 0 |
| Fungemia | 1 (1.3) | 0 | 0 |
| Hepatic infection | 0 | 1 (1.1) | 0 |
| Klebsiella infection | 1 (1.3) | 0 | 0 |
| Pseudomembranous colitis | 0 | 1 (1.1) | 0 |
| Serratia infection | 1 (1.3) | 0 | 0 |
| Sinusitis | 1 (1.3) | 0 | 0 |
| Staphylococcal bacteremia | 0 | 1 (1.1) | 0 |
| Staphylococcal sepsis | 1 (1.3) | 0 | 0 |
| Stenotrophomonas infection | 1 (1.3) | 0 | 0 |
| Streptococcal bacteremia | 0 | 0 | 1 (2.2) |
| Strongyloidiasis | 1 (1.3) | 0 | 0 |
| Systemic candida | 0 | 1 (1.1) | 0 |
| Urinary tract infection bacterial | 0 | 0 | 1 (2.2) |

BID=twice daily.

## **eTable 9. Treatment-Emergent Adverse Events With a Fatal Outcome (Safety Population)**

| **Event** | **Ruxolitinib**  **15 mg BID (n=77)** | **Ruxolitinib**  **5 mg BID (n=87)** | **Placebo (n=45)** |
| --- | --- | --- | --- |
| Any fatal treatment-emergent adverse event, n (%) | 5 (6.5) | 5 (5.7) | 5 (11.1) |
| Cardiopulmonary failure | 1 (1.3) | 1 (1.1) | 1 (2.2) |
| Cerebral hemorrhage | 0 | 2 (2.3) | 0 |
| Acute respiratory distress syndrome | 1 (1.3) | 0 | 0 |
| Cardiorespiratory arrest | 0 | 0 | 1 (2.2) |
| Myocardial infarction | 0 | 1 (1.1) | 0 |
| Pneumonia | 1 (1.3) | 0 | 2 (4.4) |
| Pneumonia staphylococcal | 1 (1.3) | 0 | 0 |
| Pneumonia bacterial | 0 | 0 | 1 (2.2) |
| Pneumonia, pathogen unspecified | 0 | 0 | 1 (2.2) |
| Pneumothorax | 0 | 0 | 1 (2.2) |
| Rectal hemorrhage | 0 | 1 (1.1) | 0 |
| Shock | 1 (1.3) | 0 | 0 |
| Staphylococcal sepsis | 1 (1.3) | 0 | 0 |

BID=twice daily.

## **eTable 10. Treatment-Emergent Adverse Events Leading to Dose Modifications (Safety Population)**

| **Event** | **Ruxolitinib**  **15 mg BID (n=77)** | **Ruxolitinib**  **5 mg BID (n=87)** | **Placebo (n=45)** |
| --- | --- | --- | --- |
| Any treatment-emergent adverse event leading to a dose reduction, n (%) | 7 (9.1) | 5 (5.7) | 2 (4.4) |
| Acute kidney injury | 2 (2.6) | 1 (1.1) | 1 (2.2) |
| Sepsis | 2 (2.6) | 0 | 0 |
| Renal failure | 0 | 2 (2.3) | 0 |
| Alanine aminotransferase increased | 0 | 1 (1.1) | 0 |
| Chronic kidney disease | 1 (1.3) | 0 | 0 |
| Hypertension | 1 (1.3) | 0 | 0 |
| Lymphocyte count decreased | 1 (1.3) | 0 | 0 |
| Pneumonia | 0 | 1 (1.1) | 0 |
| Thrombocytopenia | 1 (1.3) | 0 | 0 |
| Blood creatinine increased | 0 | 0 | 1 (2.2) |
| Pneumonia pseudomonal | 0 | 0 | 1 (2.2) |
| Streptococcal bacteremia | 0 | 0 | 1 (2.2) |
| Any treatment-emergent adverse event leading to a dose interruption, n (%) | 2 (2.6) | 4 (4.6) | 0 |
| Pneumomediastinum | 1 (1.3) | 1 (1.1) | 0 |
| Alanine aminotransferase increased | 1 (1.3) | 0 | 0 |
| Aspartate aminotransferase increased | 1 (1.3) | 0 | 0 |
| Dysphagia | 0 | 1 (1.1) | 0 |
| Hematemesis | 0 | 1 (1.1) | 0 |
| Pneumoperitoneum | 1 (1.3) | 0 | 0 |
| Thrombocytopenia | 0 | 1 (1.1) | 0 |
| Transaminases increased | 0 | 1 (1.1) | 0 |
| Any treatment-emergent adverse event leading to treatment discontinuation, n (%) | 7 (9.1) | 5 (5.7) | 1 (2.2) |
| Alanine aminotransferase increased | 2 (2.6) | 2 (2.3) | 0 |
| Aspartate aminotransferase increased | 1 (1.3) | 3 (3.4) | 0 |
| Thrombocytopenia | 1 (1.3) | 0 | 1 (2.2) |
| Anemia | 1 (1.3) | 0 | 0 |
| Bacteremia | 1 (1.3) | 0 | 0 |
| Blood alkaline phosphatase increased | 0 | 1 (1.1) | 0 |
| Cerebral hemorrhage | 0 | 1 (1.1) | 0 |
| Hypoglycemia | 1 (1.3) | 0 | 0 |
| Pneumonia | 0 | 1 (1.1) | 0 |
| Shock | 1 (1.3) | 0 | 0 |

BID=twice daily.
